# Supplementary material for: Blockade of CB1 or Activation of CB2 Cannabinoid Receptors Is Differentially Efficacious in the Treatment of the Early Pathological Events in Streptozotocin-Induced Diabetic Rats
Source: Int J Mol Sci. 2022 Dec 23;24(1):240. doi: 10.3390/ijms24010240 (PMC9820336; doi:10.3390/ijms24010240)
Supplement: Supplementary file 1 [file ijms-24-00240-s001.zip › ijms-1978765-supplementary.pdf]

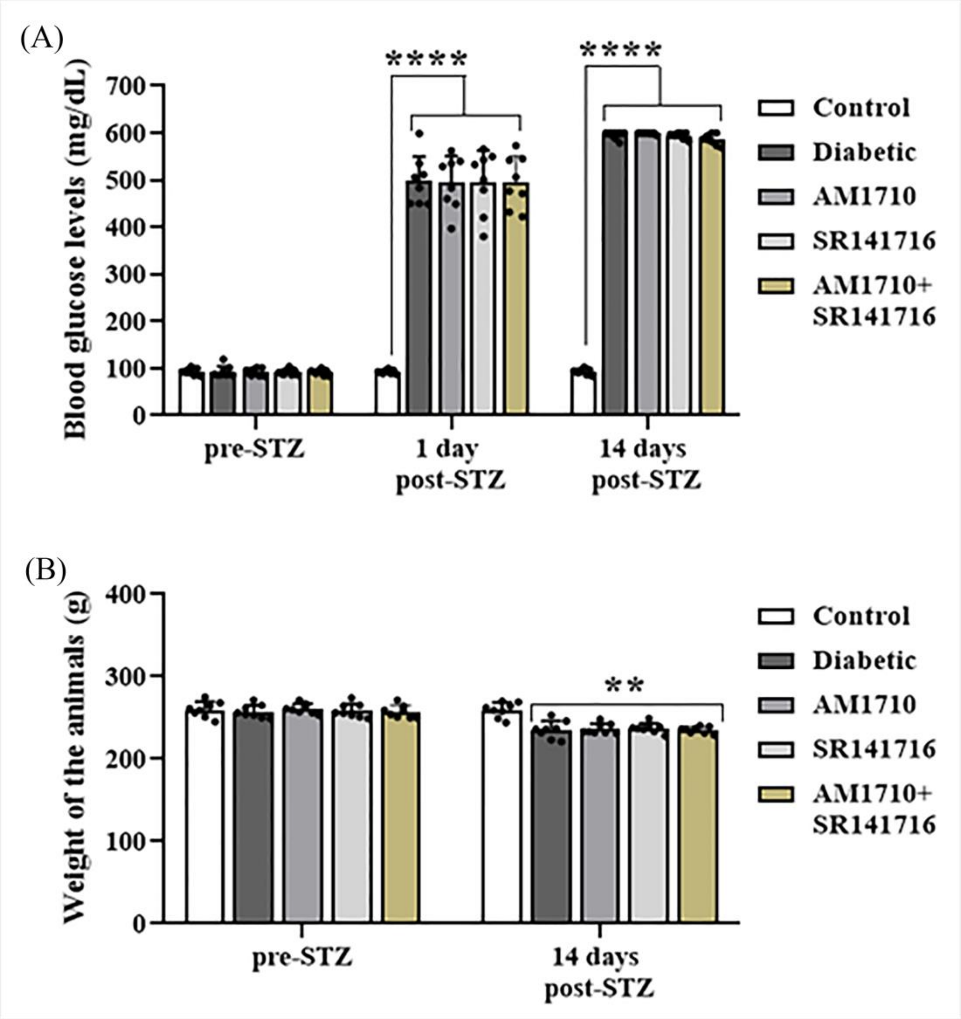

Figure S1. Effect of STZ on glucose levels and weight of experimental animals. **(A)** Measurement of blood glucose levels across the five different animal groups, before, one day and fourteen days after STZ administration. Prior to STZ administration there was no statistically significant difference in glucose levels in the five experimental groups ($p>0.05$). One day post STZ administration all diabetic animals (untreated and treated) displayed significant increases on their blood glucose compared to Control animals (****$p<0.0001$ compared to Control). Fourteen days after STZ injection, blood glucose levels of all diabetic animals remained elevated compared to Control animals (****$p<0.0001$ compared to Control). No statistically significant difference was observed on blood glucose levels among diabetic untreated and treated animals after one or fourteen days of STZ administration ($p>0.05$). **(B)** The weight of experimental animals was assessed across the five different groups, before and fourteen days after STZ administration. No difference was observed the weight among animals in the five different groups before administration of STZ. Fourteen days after STZ injection all diabetic animals experienced a reduction on their weight compared to Control animals (diabetic untreated: **$p=0.0067$; AM1710: **$p=0.0052$; SR141716: **$p=0.0023$; AM1710+SR141716: **$p=0.0045$, compared to Control). No significant difference was observed among diabetic untreated and treated animals ($p>0.05$).

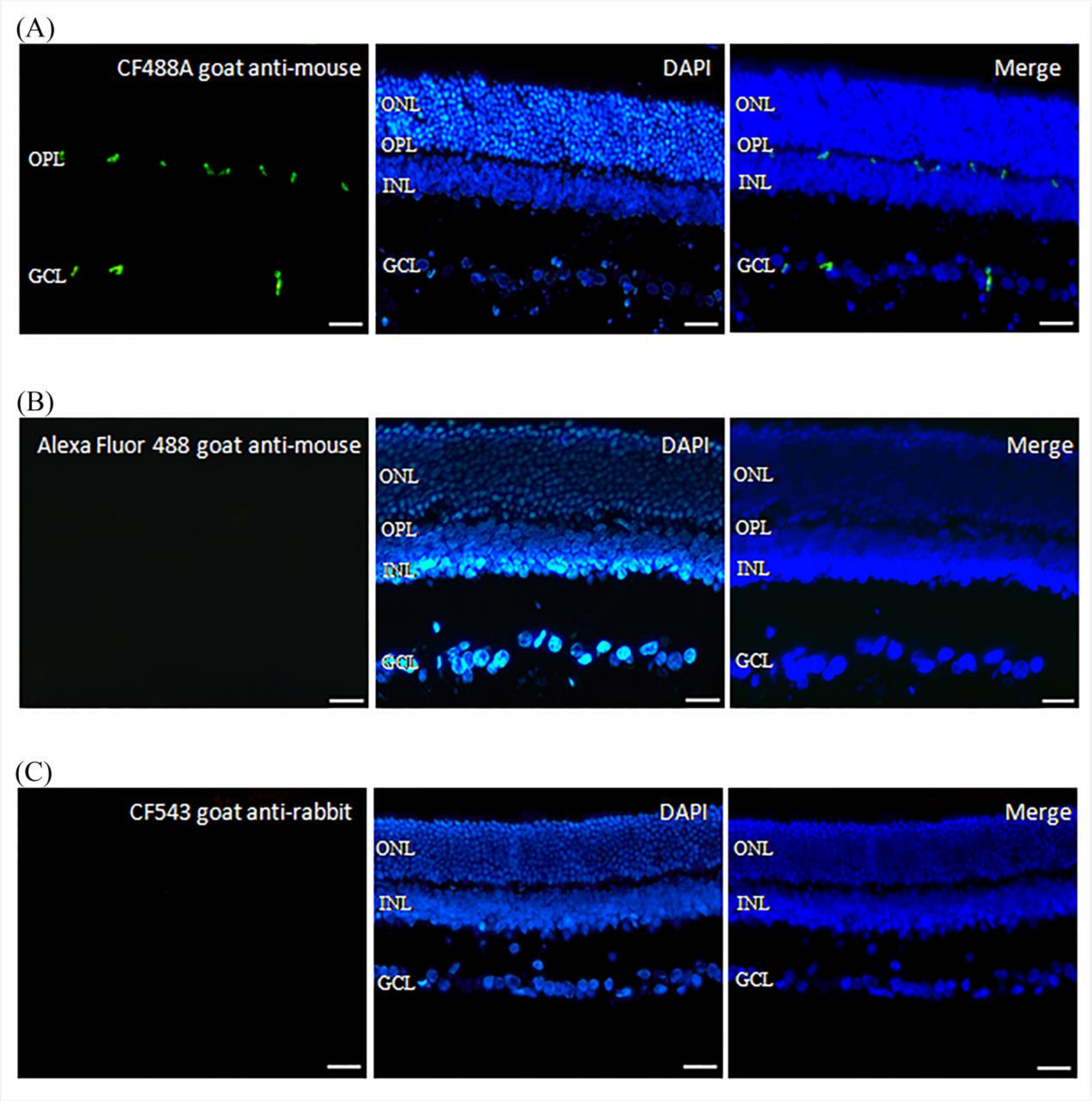

Figure S2. Representative photomicrographs of the secondary antibodies CF488A goat anti-mouse (Biotium, Fremont, CA, code no. 20010, lot no. 13C0619), Alexa Fluor 488 goat anti-mouse (Invitrogen, Waltham, Massachusetts, code no. A11029, lot no. 2306576) and CF543 goat anti-rabbit (Biotium, Fremont, CA, code no. 20309, lot no. 12C0213) with their respective nuclear staining. Magnification: 20X. Scale bar: 50 μm **(A)** CF488A goat anti-mouse displayed staining mainly on OPL and at a smaller degree on GCL. **(B)** No staining was observed with the use of Alexa Fluor 488 goat anti-mouse and **(C)** CF543 goat anti-rabbit secondary antibody. OPL: Outer plexiform layer; GCL: Ganglion cell layer.
